# An Interesting Case of Stricture of Female Urethra and Treatment

**Published:** 1879-06

**Authors:** H. L. Wilson

**Affiliations:** Atlanta, Ga.


### AN INTERESTING CASE OE STRICTURE OF FEMALE URETHRA AND TREATMENT.

By H. L. WILSON, M.D., Atlanta, Ga.

March, 1879. Was called to see a young lady, who informed me that she had been sick since last year—the latter part of the time she had been confined in bed. She had been treated, while a resident of Chattanooga, for various womb troubles, but experienced no relief. Upon reaching Atlanta she called in a physician, who pronounced her trouble cystitis, for which he prescribed. Subsequently she was treated for nephritis, uterine neuralgia, and a host of other troubles, but gradually grew worse. When I saw her she had contracted the unfortunate habit of taking opium to allay the constant pain caused by frequent micturition, which was often followed by a few drops of dark blood. She could not rest day nor night without the aid of an opiate. Her appetite was gone, resulting, of course, in emaciation. Hers was truly a pitiable condition. I made careful examination of the uterus, and to my surprise found everything in that region in a perfectly normal condition. I next proceeded to examine the urethra, and discovered that I had no instrument that I could introduce.

The next day I saw my patient, armed with a full supply of bougies. The urethra was so exceedingly sensitive that she could scarcely allow the touch of the instrument, and it was with difficulty that I reached the bladder with a No. 8 French bougie. I next introduced an olive shaped rubber bougie, and discovered a very decided and remarkable stricture half an inch posterior to the meatus. Having convinced myself that I had found the cause of all her woes, I informed her that I would operate the following day.

January 24. I introduced the meatome with considerable difficulty, carrying the blades posterior to the stricture, then withdrew the instrument with the blades well opened, making an upward cut. I next cut a little downward with a bistoury, introduced my finger, and subsequently a No. 36 bougie with perfect ease. I now withdrew the ether, which was administered by my friend, Dr. Rains, and ordered a warm hip bath, giving her at the same time sulph. quinia gr. xv. I should have administered an opiate, had I not known her opium status; but as she was then taking the opium antidote from B. M. Woolley, I left that alone.

I saw her the second day after the operation, and found her more comfortable than she had been for months. I gave more quinia, and after a hip bath introduced No. 36 bougie without the slightest trouble. She improved daily, going five to six hours without voiding urine in as many days after the operation. In two weeks she was cheerful and happy, had perfect control of her bladder, and knew that she was relieved.

Since the operation she had gained forty pounds, and to-day is the picture of health. In conversation with her during my visit, I ascertained that some four year ago she had urethritis, which, I am satisfied, was the cause of the stricture. She had some of the symptoms found in males troubled with strictures from similar causes.

